# The biosynthesis of the cannabinoids

**DOI:** 10.1186/s42238-021-00062-4

**Published:** 2021-03-15

**Authors:** M. Nazir Tahir, Farsheed Shahbazi Raz, Simon Rondeau-Gagné, John F. Trant

**Affiliations:** Department of Chemistry and Biochemistry, University of Windsor, 401 Sunset Avenue, Windsor, Ontario N9B 3P4 Canada

**Keywords:** Cannabinoid biosynthesis, Enzymatic transformation, *C. sativa*, Decarboxylation, Enzymatic mechanism

## Abstract

**Abstract:**

Cannabis has been integral to Eurasian civilization for millennia, but a century of prohibition has limited investigation. With spreading legalization, science is pivoting to study the pharmacopeia of the cannabinoids, and a thorough understanding of their biosynthesis is required to engineer strains with specific cannabinoid profiles. This review surveys the biosynthesis and biochemistry of cannabinoids. The pathways and the enzymes’ mechanisms of action are discussed as is the non-enzymatic decarboxylation of the cannabinoic acids. There are still many gaps in our knowledge about the biosynthesis of the cannabinoids, especially for the minor components, and this review highlights the tools and approaches that will be applied to generate an improved understanding and consequent access to these potentially biomedically-relevant materials.

**Graphical abstract:**

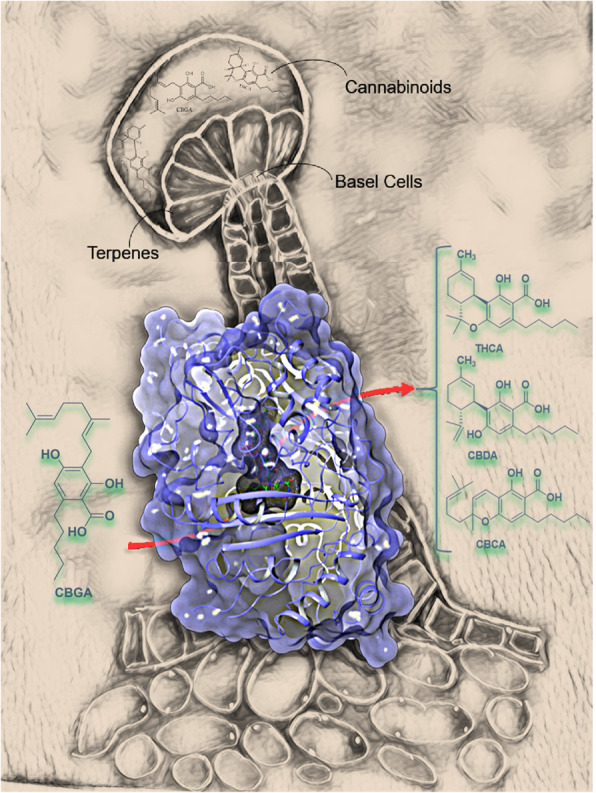

**Supplementary Information:**

The online version contains supplementary material available at 10.1186/s42238-021-00062-4.

## Main text

### Historical context of cannabis science

*Cannabis sativa* L. (*C. sativa*, see glossary) has been a staple of Eurasian culture. It is documented in Chinese texts since before 2000 B.C. (Russo 2014); the Hindu *Atharvaveda*, composed between 1500 and 1000 B.C., where it is revered as a sacred plant for ritual and ceremony (Russo 2005); and in New Kingdom Egyptian texts dating to 1550 B.C. (Hallmann-Mikolajczak 2004). Today, *C. sativa* is openly cultivated in more than 86 countries in Africa, the Americas, Asia, and Europe (United Nations Office on Drugs and Crime 2005), although the number is likely far higher. However, its chemical characterization history is far briefer. Δ^9^-Tetrahydrocannabinol (THC), the primary psychoactive component, was first isolated as an impure resin in 1942, when the structure was proposed (Wollner et al. 1942). In 1955, the first cannabinoid, cannabidiolic acid (CBDA), was isolated in a pure form by Krejčí and Šantavý. As recently as the late 1960s, it was considered that the active principles of cannabis were an unidentified mixture of tetrahydrocannabinols (Mechoulam 1970). A crystallizable Δ^9^-THC derivative was prepared in 1964, allowing ready access to the parent compound and ensuring that its structure and stereochemistry were correctly assigned (Gaoni and Mechoulam 1964), with CBD following soon after (Šantavý 1964). The literature contains a series of foundational reviews covering the chemistry of cannabinoids that contextualize the advancement of the field. Farnsworth’s 1969 review covers botanical considerations of “marihuana,” the biological evaluation of the plant and extracts, the known chemical constituents, and the method for their identification (Farnsworth 1969). Mechoulam’s 1970 influential review on “marihuana chemistry” discussed nomenclature and the chemical and the then-proposed biogenic synthesis of cannabinoids (Mechoulam 1970). In 1975, Shoyama et al. in their “biosynthesis of cannabinoid acids” discussed potential pathways used to synthesize cannabinoic acids from cannabigerolic acid (Shoyama et al. 1975). Turner and coauthors discuss the natural constituents and classes of metabolites of *C. sativa* in their 1980 review (Turner et al. 1980). However, the field has progressed quickly in the decades since, rendering these more of historical interest for the evolution of our understanding of cannabis. The most recent review on cannabis biosynthesis was authored by Flores-Sanchez and Verpoorte in 2008, describing the biosynthesis of all major secondary metabolites of *C. sativa*, e.g., flavonoids, stilbenoids, terpenoids, alkaloids, lignanamides, and phenoic amides in addition to the cannabinoids (Flores-Sanchez and Verpoorte 2008). The field has evolved significantly in the 12 years since, especially in a complete revaluation of the synthesis of olivetolic acid, the common precursor of the cannabinoids (Taura et al. 2009). This review complements recent reviews on cannabinoid structural biology (Shahbazi et al. 2020), cannabinoid biological activity (Kinghorn et al. 2017), and an excellent short introductory review to cannabis science from Reekie, Scott, and Kassiou (2017).

Cannabinoids are prenylated polyketides produced in *C. sativa* (Taura et al. 2007a). More than 1600 chemical compounds have been isolated from *C. sativa*, of which over 180 are cannabinoids (Hanuš et al. 2016) that can be classified into 11 structural families (Table S1) (Kinghorn et al. 2017; Taura et al. 2007a; Zirpel et al. 2018; Merlin 2003). The most abundant cannabinoids (naturally present as their corresponding carboxylic acid) are Δ^9^-tetrahydrocannabinol (Δ^9^-THC or THC, the main psychoactive cannabinoid), cannabidiol (CBD), and cannabichromene (CBC); these are supplemented by other classes archetyped by Δ^8^-trans-tetrahydrocannabinol (Δ^8^-THC), cannabigerol (CBG), cannabinodiol (CBND), cannabielsoin (CBE), cannabicyclol (CBL), cannabinol (CBN), cannabitriol (CBT), and a miscellaneous group (Kinghorn et al. 2017; Taura et al. 2007a; Merlin 2003; Hampson et al. 1998; Lastres-Becker et al. 2005). In this review, we will focus on the biosynthesis of major cannabinoids, i.e., THC, CBD and CBC, as the best studied, both in terms of biosynthesis and pharmacological properties (Scheckel et al. 1968; Pertwee 2008; Pertwee 2005; FDA, FDA and Cannabis: Research and Drug Approval Process (n.d.); Petrosino et al. 2018; Lewis et al. 2017). For our limited knowledge about the biosynthesis of other cannabinoids, readers are referred elsewhere (Ferioli et al. 2000; Husni et al. 2014; Radwan et al. 2015; Wakshlag et al. 2020), with the caveat and encouragement that much work remains to be done in better understanding these biochemical pathways. Similarly, readers interested in biotechnological synthesis of cannabinoids are referred to Luo et al. (2019), and to Leahy and coworkers for a clever example of their chemical synthesis (Shultz et al. 2018). The biosynthesis of the cannabis terpenes, present in small amounts in *C. sativa* (Hillig 2004; Fischedick et al. 2010; Booth et al. 2017; Booth and Bohlmann 2019; Mudge et al. 2019; Zager et al. 2019) and proposed to synergistically accentuate the pharmacological effects of cannabis consumption (Livingston et al. 2020; Russo 2011), is described elsewhere (Flores-Sanchez and Verpoorte 2008; Page et al. 2006).

**Fig. 1:**
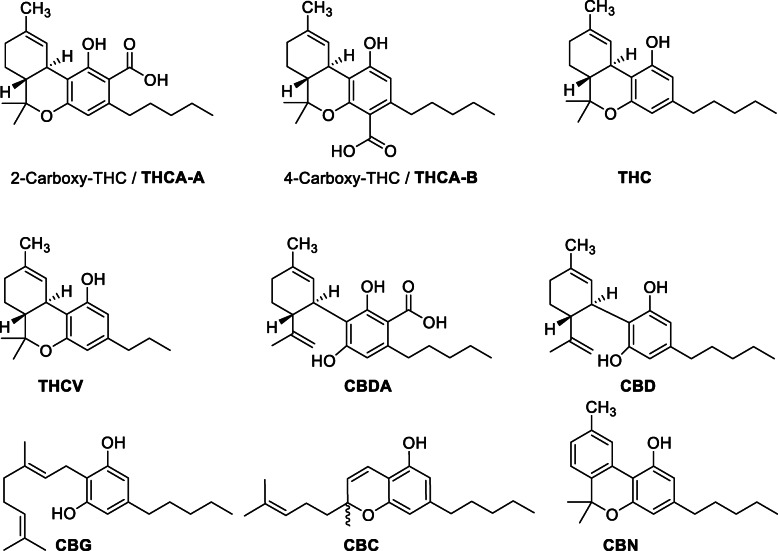
Structures of important *Cannabis sativa* cannabinoid classes: Δ^9^-tetrahydrocannabinol (Δ^9^-THC or THC) and its acidic counterparts (THCA-A/THCA-B), tetrahydrocannabivarin (THCV), cannabidiol (CBD) and its acidic counterpart (CBDA), cannabigerol (CBG), cannabichromene (CBC), and cannabinol (CBN)

In *C. sativa*, cannabinoids are biosynthesized as phytoprotectants: in fresh biomass, 95% of the THC, CBD, and CBC exist as their acidic parents: tetrahydrocannabinolic acid (THCA), cannabidiolic acid (CBDA), and cannabichromenic acid (CBCA) (United Nations Office on Drugs and Crime 2005). These decarboxylate to the more familiar forms during storage, upon heating, or under alkaline conditions (United Nations Office on Drugs and Crime 2005; Ghosh et al. 1940; Adams et al. 1940; Taura 2009). “Tetrahydrocannabinolic acid” or “THCA” has been used vaguely and can refer to several constitutional isomers, making physiological and pharmacological profiling confusing (Moreno-Sanz 2016). In 1965, Korte et al. (1965) identified *tetrahydrocannabinolcarboxylic acid* (2-carboxy-THC, Fig. 1) as a major component of hashish. In 1969, Mechoulam reported on a second THC acid isomer, the 4-carboxy-THC (Fig. 1), and named Korte’s THCA-A and his THCA-B (Mechoulam et al. 1969). THCA-B was only found in hashish samples with little to no THCA-A, and its overall concentration was generally lower than 0.5 weight percent. Subsequent studies, however, were not able to confirm the occurrence of THCA-B (De Zeeuw et al. 1972). Therefore, in this review, we will use only the term THCA and it always refers to THCA-A (2-carboxy-THC, Fig. 1) unless stated otherwise.

The *Cannabis* genus comprises three species defined by their phytocannabinoid content: the low-THC *C. sativa* L., the high Δ^9^-THC, *C. indica* Lam., and an intermediate species, *C. ruderalis* Janisch (Mechoulam 1970; Hartsel et al. 2016; Appendino et al. 2011; Thomas and ElSohly 2016); however, as the three species readily interbreed and many existing cultivars are hybrids, a monotypic classification, *C. sativa*, is gaining traction with subdivisions into chemotypes rather than species (Pellati et al. 2018). Varieties used for drug consumption, characterized by a high content of Δ^9^-THC, are often not morphologically distinguishable from low-THC fiber-type varieties. Biosynthesis proceeds through the same pathways in all species.

### Cannabinoids are synthesized through a common pathway in trichomes

The cannabinoids are biosynthesized in the glandular trichomes, or “marijuana bud” of female flowers; trichome-poor male flowers are typically very low in cannabinoids (Livingston et al. 2020). Trichomes are also present on bracts, leaves, and on the underside of the anther lobes of male flowers (Mahlberg et al. 1980). *Trichōma*, Greek for “hair” (Figure S1a) (Kenneth 2018), are classified as stalked, sessile, or bulbous (Figure S1 b-d) (Hammond and Mahlberg 1973). Bulbous trichomes, the smallest in size, produce limited cannabinoids; the other two morphologies are responsible for almost all cannabinoid production. Sessile trichomes, supported by a short stalk, have a globose head comprising a multicellular disc of secretory cells with a subcuticular metabolite storage cavity (Hammond and Mahlberg 1977). Stalked trichomes have a slightly larger globose head, rising several hundred microns above the epidermal surface (Mahlberg and Kim 2004). The relative contribution of sessile and stalked trichomes to cannabinoid production remains unclear (Livingston et al. 2020).

**Fig. 2:**
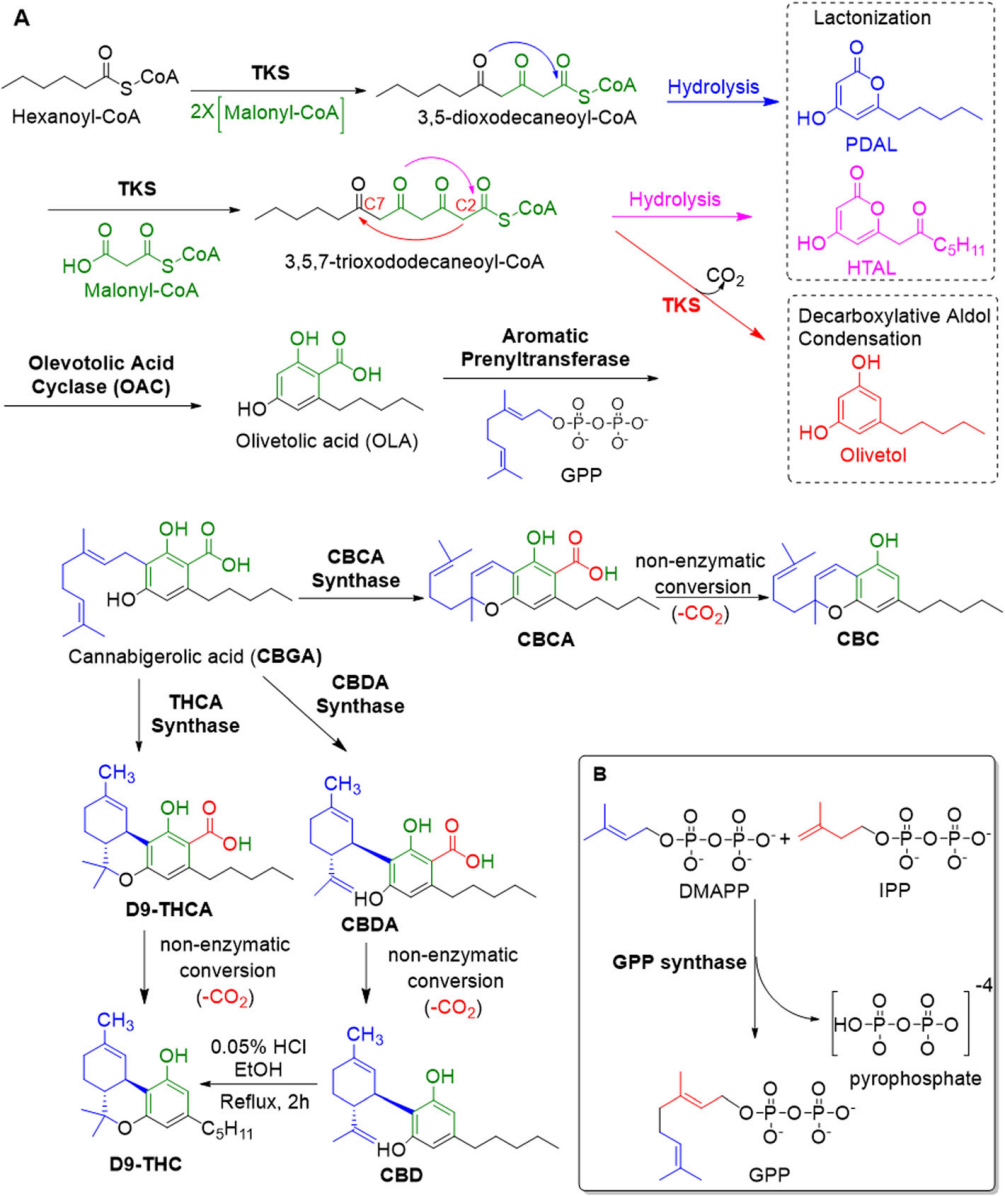
Biosynthesis of cannabinoids. **a** Proposed cannabinoid biosynthetic pathway for Δ^9^-tetrahydrocannabinol (Δ^9^-THC), cannabidiol (CBD), and cannabichromene (CBC) including by-product formation (pentyl diacetic lactone (PDAL), hexanoyl triacetic acid lactone (HTAL), and olivetol shown in the dotted box) and highlighting the chemical conversion of CBD into THC, long thought to be the source of THC, but this conversion does not occur in vivo. **b** Synthesis of geranyl pyrophosphate (GPP) from dimethylallyl pyrophosphate (DMAPP) and isopentenyl pyrophosphate (IPP) catalyzed by geranyl pyrophosphate synthase

The biosynthesis of cannabinoids remains incompletely understood at the molecular level (Fellermeier and Zenk 1998). In brief, cannabinoids share a common initial pathway: tetraketide synthase (TKS) (Kearsey et al. 2020), a type III polyketide synthase (PKS), catalyzes the sequential condensation of hexanoyl-CoA with three molecules of malonyl-CoA to yield 3,5,7-trioxododecaneoyl-CoA (Fig. 2a) (Taura et al. 2007b). This is cyclized and aromatized, with the loss of Coenzyme A, by olivetolic acid cyclase (OAC), to olivetolic acid (OLA) (Gagne et al. 2012). Aromatic prenyltransferase then inserts the prenyl group at the highly nucleophilic 2-resorcinol position to provide cannabigerolic acid (CBGA) (Fellermeier and Zenk 1998). This core intermediate then diverges to provide the cannabinolic acids (THCA, CBDA, and CBCA) that proceed to THC, CBD, and CBC by non-enzymatic decarboxylation (Fig. 2a) (Flores-Sanchez and Verpoorte 2009).

### Olivetol synthase and olivetolic acid cyclase cooperate to deliver the key intermediate

Olivetolic acid (OLA), forms the polyketide nucleus of the cannabinoids (Taura et al. 2009; Gagne et al. 2012; Tan et al. 2018). TKS was long thought to be solely responsible for OLA biosynthesis, with spontaneous cyclization and aromatization occurring following the addition of the third malonyl group as shown in the second step of Fig. 2a. However, while investigating the role of the enzyme, Taura and co-workers used a cDNA, encoding olivetol synthase (OLS) cloned from *C. sativa*, and found that their recombinant OLS did not produce OLA, but only its decarboxylated form, olivetol (Fig. 2a) (Taura et al. 2009). The authors also confirmed that crude enzyme extracts of either flowers or early-growth leaves, the two major cannabinoid-producing tissues of *C. sativa*, also only provided olivetol (Taura et al. 2009; Dewick 2002). This strongly indicated that OLA biosynthesis is not dependent on OLS alone, but may involve other enzymes; however, they considered that olivetol may be an artifact of in vitro enzyme assays as olivetol is not detected in *C. sativa* (Taura et al. 2009). This conundrum, OLA cannot be prepared in vitro, but the in vitro product, olivetol, is not observed in vivo, has since been resolved by evidence that the process requires olivetolic acid cyclase (OAC), to conduct the intramolecular C2 → C7 aldol condensation without decarboxylation (Fig. 2a) (Gagne et al. 2012; Tan et al. 2018; Kearsey et al. 2019). Kearsey et al. (2019) also confirmed that, in the absence of OAC, a nonenzymatic C2 → C7 decarboxylative aldol condensation of the tetraketide intermediate occurs forming olivetol instead of OLA (Fig. 2a). Non-enzymatic background cyclization generates olivetol (Austin et al. 2004). The cyclase ensures that the carboxylate survives biosynthesis.

**Fig. 3:**
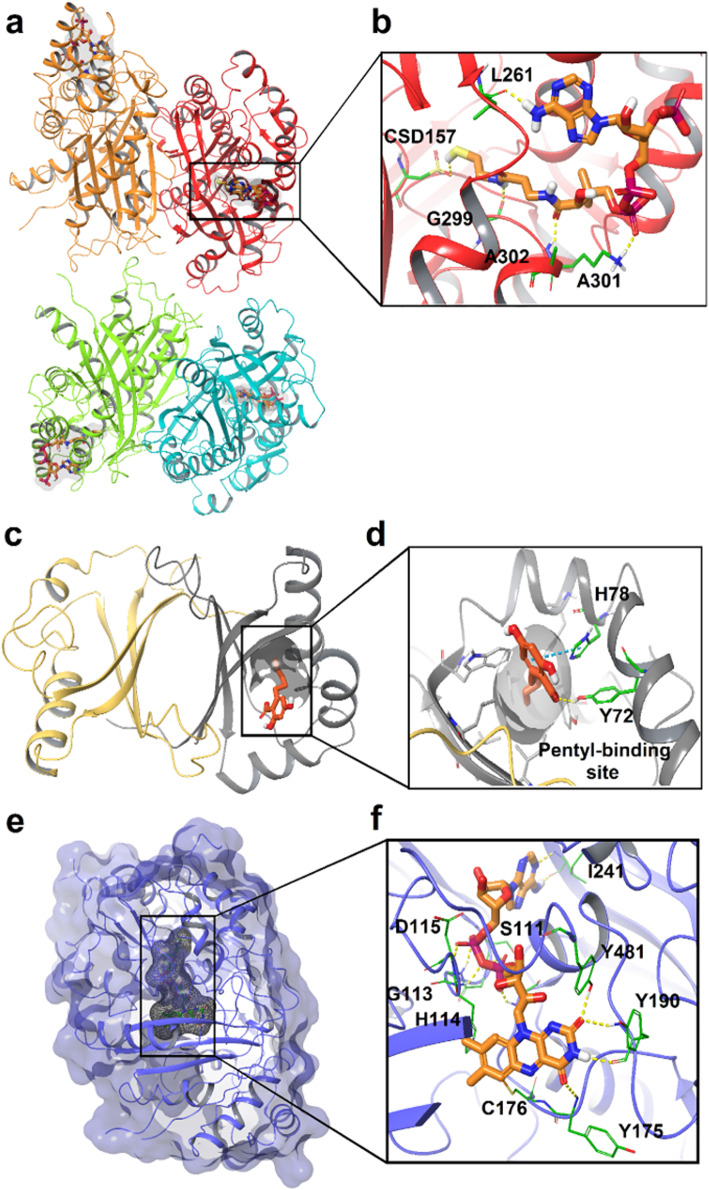
X-ray diffraction structures of three key enzymes implicated in cannabinoid biosynthesis. Key active site residues are highlighted in green, and interaction diagrams, generated by the authors using the Schrödinger computational software suite (Maestro 2020), of **a**) tetrameric tetraketide synthase (TKS) from *C. sativa* in complex with CoenzymeA (CoA, *6GW3*), CoA is orange, with the four tetramers in red, orange, light green and cyan respectively (Kearsey et al. 2020); **b**) expansion of the active site; **c**) Olivetolic acid cyclase (OAC) from *C. sativa* (*5BO9*) (Yang et al. 2016), the pentyl-binding pocket and its key residues are gray, olivetolic acid (OLA) is orange, chain A is gray and chain B is in light orange; **d**) expansion of the active site, inverted; **e**) tetrahydrocannabinolic acid synthase (THCAS) from *C. sativa* (*3VTE*) bound to flavin adenine dinucleotide (FAD) and without ligand. FAD is orange and the protein navy; and **f**) expansion of the active site (Shoyama et al. 2012)

This raises questions, however, as OLS does not interact with OAC, so the metabolite is not directly transferred, rather it must diffuse from one enzyme to the other through the cytosol (Tan et al. 2018). Kearsey et al. also investigated the crystal structure of TKS in the presence of CoA (Fig. 3a) and also performed a structure-guided mutagenesis study to investigate why the tetraketide intermediate is released prior to OAC-free cyclization (Kearsey et al. 2019). Noel and co-workers had suggested that an ‘aldol switch’ is necessary to trigger tetraketide release, thereby enabling subsequent olivetolic acid production catalyzed by OAC (Austin et al. 2004). However, Kearsey’s work does not support the presence of a universal or predictable ‘aldol switch’ consensus sequence. During the formation of OLA, small quantities of pentyl diacetic lactone (PDAL) and hexanoyl triacetic acid lactone (HTAL) are also formed from non-enzymatic hydrolysis of the mono- and di-malonylated intermediates respectively (Fig. 2a) (Taura et al. 2007b; Gagne et al. 2012; Kearsey et al. 2019).

OLA is then converted into cannabigerolic acid (CBGA) through the addition of geranyl pyrophosphate (GPP) catalyzed by an aromatic prenyltransferase (APT) (Lercker et al. 1992). GPP is synthesized by the condensation of isopentenyl pyrophosphate (IPP) and dimethylallyl pyrophosphate (DMAPP) catalyzed by geranyl pyrophosphate synthase (Fig. 2b) (Davis and Croteau 2000; Bohlmann and Gershenzon 2009 ). CBGA then converts to THCA, CBDA, and CBCA (Fig. 2a) (Tan et al. 2018; Shoyama et al. 2012).

In 1960s and 1970s, numerous plausible hypotheses have been advanced regarding the biosynthesis of THCA; however, they all were lacking experimental support. THCA was thought to arise from CBDA through cyclization (Mechoulam 1970; Gaoni and Mechoulam 1964; Shoyama et al. 1975; Taura 2009). This was endorsed by Gaoni and Mechoulam, who, while establishing the structures of CBD and THC, boiled CBD with 0.05% v/v HCl in ethanol for 2 h, obtained a mixture of THC and starting material (Fig. 2a) (Gaoni and Mechoulam 1964). However, the reaction conditions of the transformation do differ from those present during natural biosynthesis in the plants; moreover, isomerase activity, which would be necessarily responsible for the conversion of CBDA into THCA, has never been detected in any enzyme assays using crude *C. sativa* enzyme extracts. Current thinking suggests it comes from CBGA instead by either tetrahydrocannabinolic acid synthase (THCAS) or cannabidiolic acid synthase (CBDAS), both members of the *p*-cresol methyl-hydroxylase super family (Taura et al. 2007b; Shoyama et al. 2012).

### Structural and mechanistic nature of TKS, OAC, THCAS, and CBDAS

The crystal structure of TKS has recently been released by Kearsey et al. in 2019 (PDBID: *6GW3*) (Fig. 3a) (Kearsey et al. 2020). Two dimers are involved in the asymmetric unit and there is no significant difference in conformation between the four monomers. The resident CoA forms five hydrogen bonds with residues CDS157, LEU261, GLU299, LYS301, and ALA302 (Fig. 3a). The active site is reasonably flexible to be compatible with a growing polyketide substrate. The CoA ligand sits with the sulfur atom near the catalytic Cys157, which was oxidized to the sulfinic acid during crystallization. The putative catalytic water molecule is coordinated to both Ser332, CSD157 and also interacts with other water molecules.

Although the crystal structure of the full protein is unavailable, structural data from a truncated OAC and its OAC–OLA binary complex, existing as a homodimer, has been reported by Yang et al. (Fig. 3b) (Yang et al. 2016). OAC’s active-site cavity incorporates 18 residues; nine of them form a long hydrophobic tunnel, the pentyl-binding pocket, deep inside the active-site cavity to selectively accommodate OLA’s pentyl chain (Fig. 3b), ensuring OLA’s dihydroxy-benzoate moiety sits at the entrance of the active-site cavity. OAC’s Tyr72 and His78 form H-bond and π-π interactions respectively with OLA, and also act as the acid and base catalysts to assist cyclization.

**Fig. 4:**
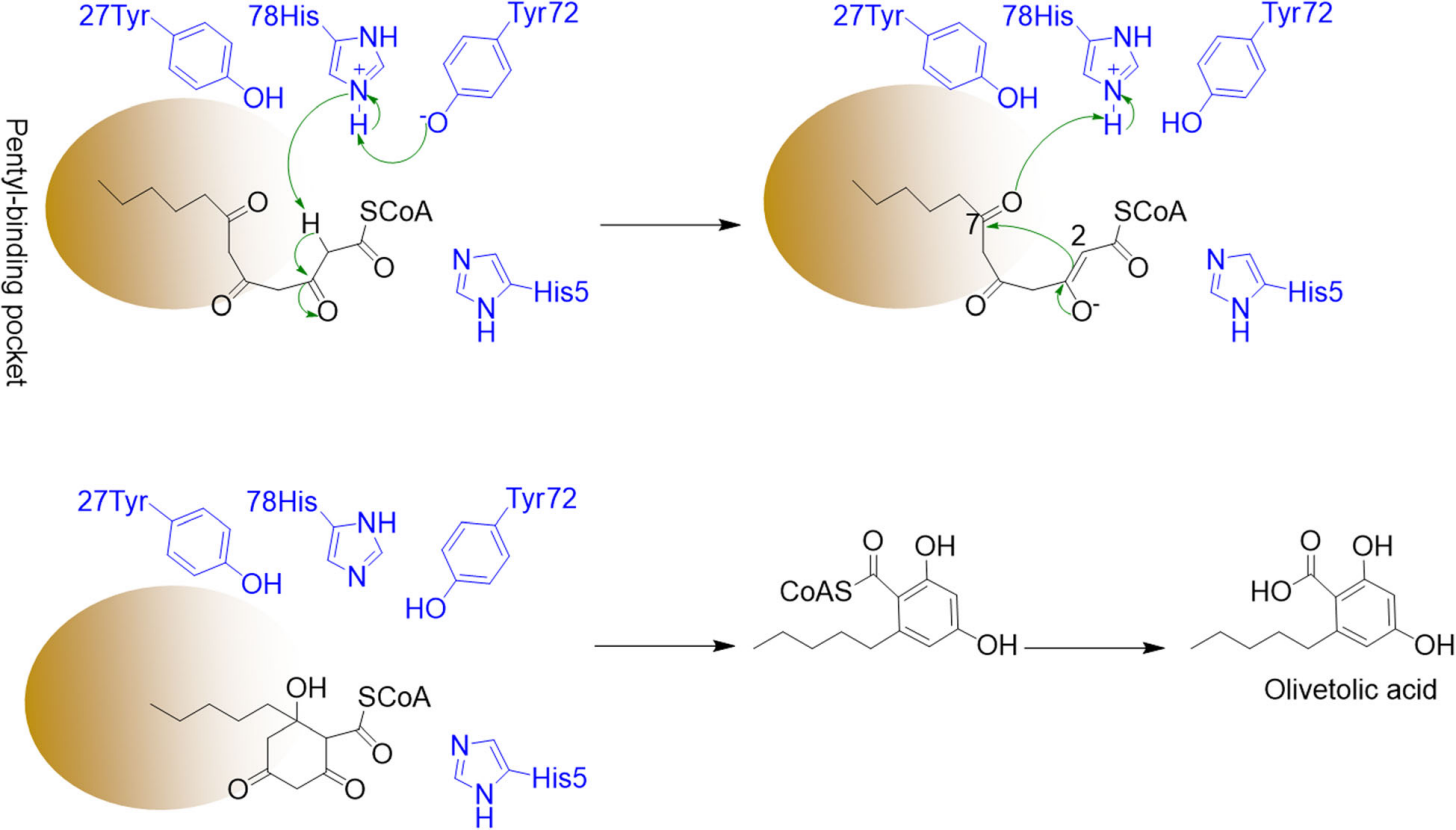
Proposed mechanism for the formation of olivetolic acid (OLA) by olivetolic acid cyclase (OAC)

Docking studies of the pentyl tetra-β-ketide CoA into the OAC structure portended that His78 and Tyr72 are involved in the catalytic mechanism. Yang et al*.* (2016) proposed that His78 deprotonates the C2 carbon of 4.1, and then protonates of the C7 oxygen in 4.2 to catalyze the desired aldol cyclization to 4.3 (Fig. 4). Tyr72 activates the side chain of His78 (through deprotonation) and the thioester carbonyl oxygen of the substrate (through hydrogen bonding). No residues, metal ions, or water molecules that may be involved in the thioester bond cleavage and aromatization were observed in the OAC–OA binary complex structure. This suggested that OAC lacks both thioesterase and aromatase activities. OAC consequently employs standard acid/base catalytic chemistry for the formation of precursor 4.3, which then dissociates from the enzyme and aromatizes and hydrolyzes to provide OLA (Yang et al. 2016).

In 1995, Taura and co-workers experimentally identified a new 76 kDa monomeric oxidoreductase, THCAS, that converts CBGA into THCA (Taura 2009; Taura et al. 1995) when CBGA was treated with an extract from young leaves, high levels of THCA were produced. The Taura group produced a cDNA sequence to simplify its study through heterologous expression, the first enzyme involved in cannabis biosynthesis to be cloned The parent gene THCAS consists of a 1635-nucleotide open reading frame, encoding a 545-amino acid polypeptide, the first 28 of which constitute the signal peptide (Sirikantaramas et al. 2004). As an extension of this effort, they developed a fermentation-friendly expression system for THCAS, a requirement for the biotechnological production of Δ^9^-THC (Sirikantaramas et al. 2004; Taura et al. 2007c). This vision has been fully realized by the recent work of Keasling that allows for the access of cannabinoids from yeast (Luo et al. 2019).

**Fig. 5:**
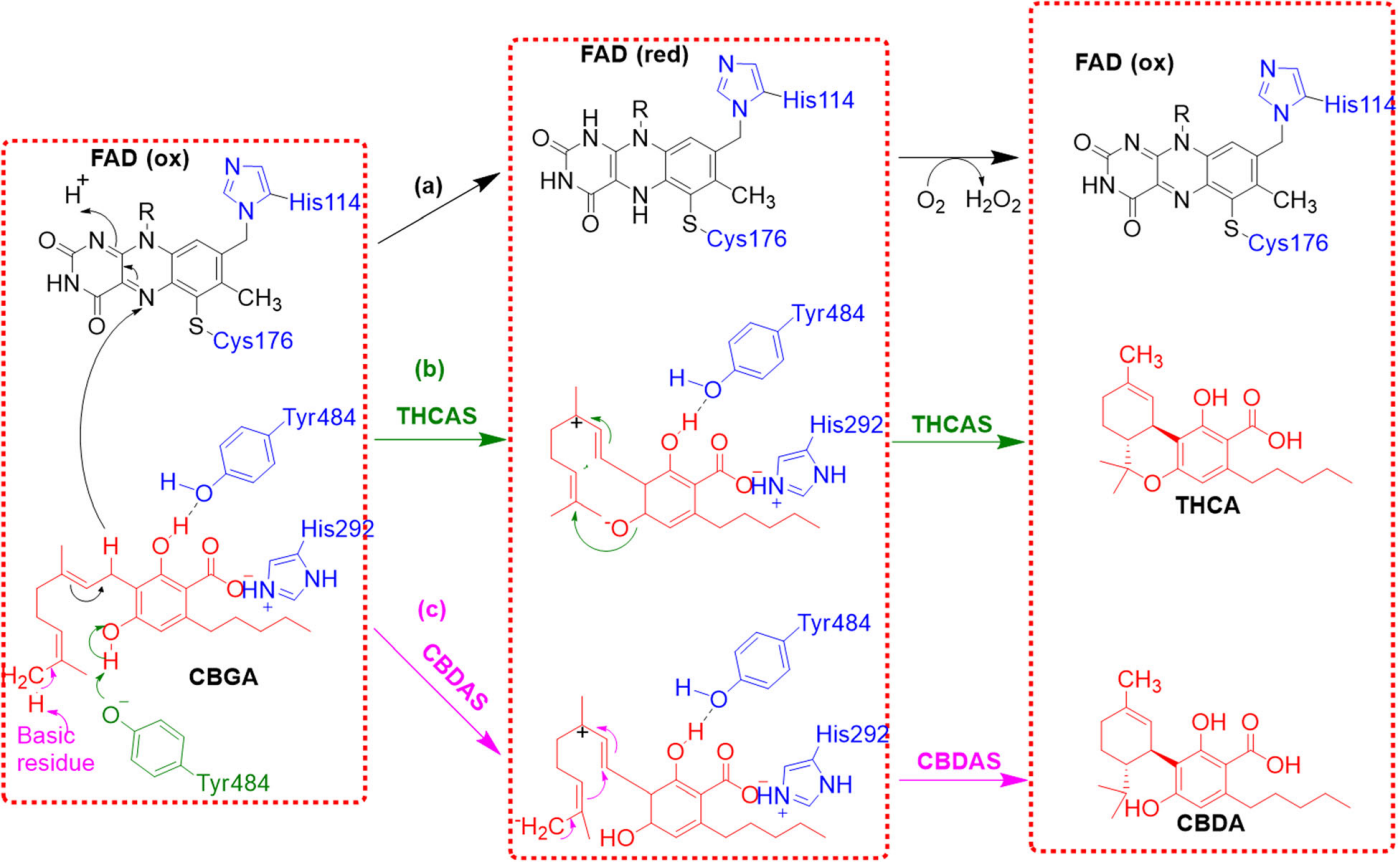
Reaction mechanism for the conversion of cannabigerolic acid (CBGA) into tetrahydrocannabinolic acid (THCA) proposed by Taura and cowerkers (2019). **a** Covalently incorporated flavin adenine dinucleotide (FAD) in black, **b** THCA synthase pathway is shown in green, and **c** cannabidiolic acid (CBDA) synthase pathway is shown in purple; CBDA and THCA in red. The red box represents the active site of the enzyme

In 2012 Kuroki and Morimoto reported an X-ray crystal structure of THCA synthase that provides significant mechanistic insight: the active site locks FAD in place through two covalent bonds with His114 and Cys176 (Fig. 3c) (Shoyama et al. 2012). This covalent immobilization is supported by a series of key H-bonds with 10 additional residues, making FAD a permanent feature of the enzyme; this ligand, along the Cys37-Cys99 disulfide bridge, drives proper folding of the rest of the active site. These combine to immobilize CBGA to facilitate hydride transfer to FAD setting up a formal enantiospecific hetero Diels-Alder reaction (Zirpel 2018), although the mechanism likely proceeds through a standard carbocation ionic pathway (Fig. 5a).

CBDAS is a 517-amino acid polypeptide with a theoretical mass of 59 kDa, although no crystal structure has been obtained (Taura et al. 2007b; Lercker et al. 1992). Experimentally, it has been detected as a 74 kDa protein, possibly the result of posttranslational N-glycosylation of seven Asn residues (Taura et al. 2007b; Taura et al. 1996). Like THCAS, CBDAS is also a flavinated enzyme; His114 and Cys176 are the most likely FAD-binding sites based on analogy with THCAS. Morimoto has proposed that the mechanism of the two enzymes is likely very similar (Taura et al. 2007b). The Morimoto group has proposed that the significant difference between their primary mode of action is in the proton transfer step: CBDAS abstracts a proton from the terminal methyl group of CBGA instead of from the hydroxyl group targeted by THCAS, this change in regioselectivity determines the cyclization (Fig. 5b, c) (Taura et al. 2007b; Taura et al. 2019).

**Fig. 6:**
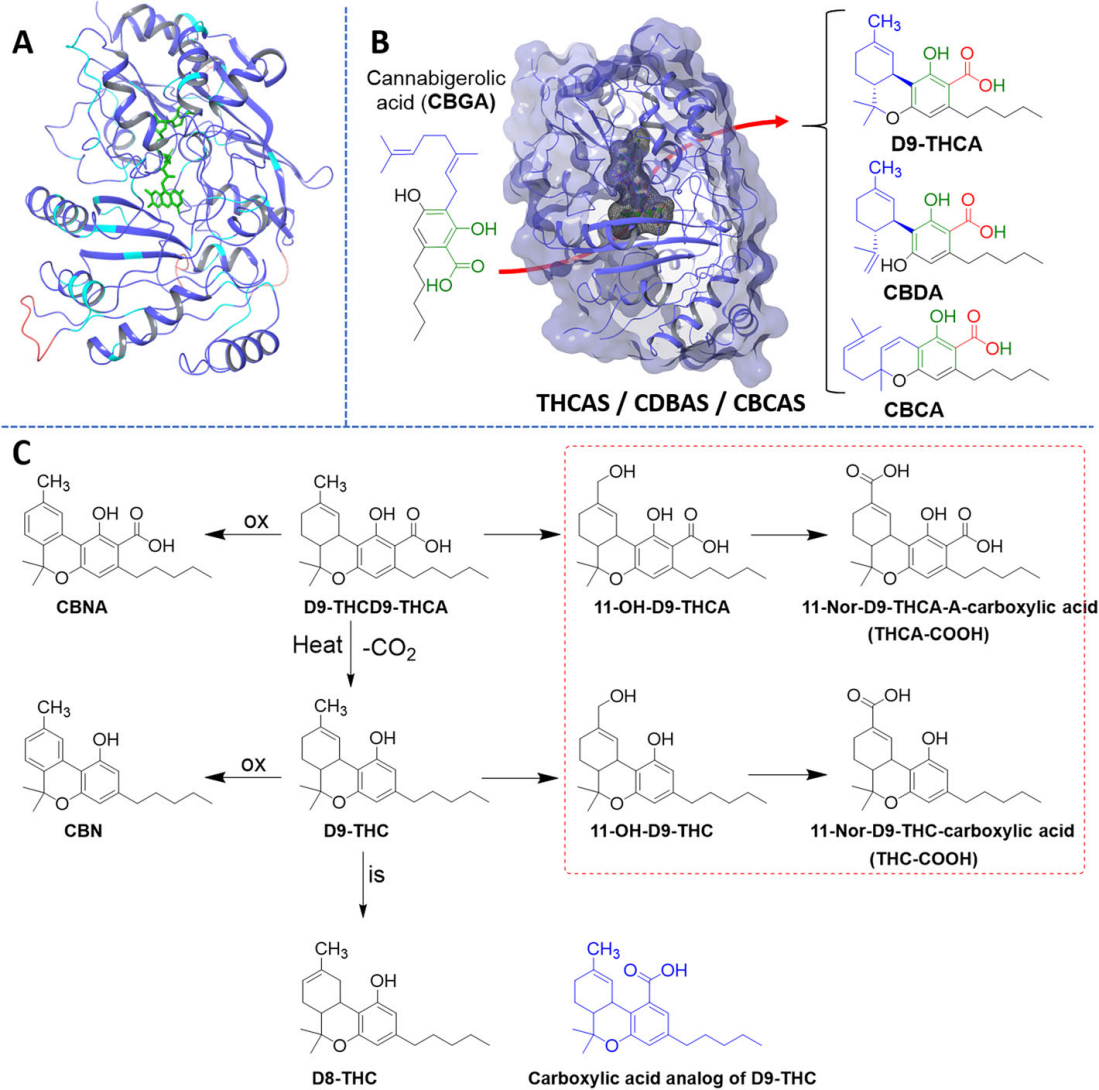
Comparison of cannabidiolic acid synthase (CBDAS) and tetrahydrocannabinolic acid synthase (THCAS) and the metabolism of cannabinoids. **a** Homology model of CBDAS developed from THCAS (*3VTE*); residues conserved from THCAS are purple while variant residues are cyan, sequence insertions are red, and FAD is green; **b** active site of these enzymes highlighted with a cartoon showing conversion to tetrahydrocannabinolic acid (THCA), cannabidiolic acid (CBDA) and cannabichromenic acid (CBCA) from cannabigerolic acid (CBGA); **c** experimentally demonstrated oxidation (ox) and isomerization (is) reactions and metabolic fates (encircled) for Δ^9^-THCA and Δ^9^-Tetrahydrocannabinol (Δ^9^-THC)

Despite this minor difference in mechanism, THCAS and CBDAS have 84% sequence identity (Taura et al. 2007b), with mutations at key active site residues likely explaining their differing cyclization specificity (Fig. 6a) (Onofri et al. 2015). they both generate eight different products, although in different ratios. Whereas THCAS produces CBDA and CBCA as minor products, CBDAS produces small amounts of THCA and CBCA in addition to CBDA (Fig. 6b) (Zirpel et al. 2018). This similarity can be exploited and a simple point mutation, A414V in THCAS yields an analog with threefold higher catalytic activity for the production of CBDA than THCAS, but also with 19-fold higher production of THCA and a broadened pH spectrum for the production of CBDA, THCA, and CBCA (Zirpel et al. 2018).

Recently the sequence of CBCAS was reported by Page and Stout (2019). The sequence identity between CBDAS and THCAS is near identity: 96% (van Bakel et al. 2011). Morimoto had previously purified the enzyme to apparent homogeneity, but this sequence is not yet available in public databases (Morimoto et al. 1998). CBCAS was isolated and partially purified from young leaves of *C. sativa* (Morimoto et al. 1998; Morimoto et al. 1997). In contrast to CBDAS and THCAS, CBCAS seems to be homodimer with a determined native molecular mass of 136 kDa and a maximum activity at pH 6.5. A molecular mass of 71 kDa was estimated for the monomers using SDS-PAGE. According to kinetic data, CBCAS has a higher affinity for CBGA than THCAS and CBCAS (Morimoto et al. 1998). CBCA and its neutral form CBC are both racemic. Studies by Morimoto suggested that both enantiomers of CBCA are formed by a CBCAS catalyzed reaction in a molar ratio of 5:1 (Morimoto et al. 1997). But it is still unknown which of the two isomers is the major product (Taura et al. 2007a; Morimoto et al. 1997; Gaoni and Mechoulam 1971). Much work remains to be done to better understand this enzyme.

### Decarboxylation of cannabinoid acids

The neutral cannabinoids, like Δ^9^-THC and CBD do not occur at significant concentrations in the plants but are readily accessed by nonenzymatic thermal decarboxylation when exposed to light or heat via smoking or baking (Tan et al. 2018)^.^ To characterize decarboxylation, sensitive analytical methods are needed to quantify, in real-time, the concentrations of both acids and neutral cannabinoids in their complex matrix (Wang et al. 2016). Temperature and heating duration are very important: over-heating directly decomposes cannabinoids and prolonged reaction times induce side reactions including over-oxidation, decreasing the yield and increasing the impurity profile (Fig. 6c). Chemical analyses are usually reported as the sum of the acidic and neutral forms of the cannabinoids; furthermore, THC levels are reported as a combination of THC and CBN levels as Δ^9^-THCA and Δ^9^-THC themselves readily oxidize respectively to CBNA and cannabinol (CBN, Fig. 6c) with heat, oxygen, and light (Moreno-Sanz 2016; Pellati et al., 2018; Dussy et al. 2005). These levels are measured primarily using either gas or liquid chromatography (GC and LC) (Wang et al. 2016). Based on the work of many analytical studies using gas and liquid chromatography over recent years (for a detailed review of the contributions of various authors, please see the SI, Figures S2 and S3), the current proposed mechanism for thermal decarboxylation invokes an intramolecular hydrogen bond with the *ortho*-phenol (Figure S4) and appears to be a commonality for this series of 2-hydroxybenzoic acids (Perrotin-Brunel et al. 2011).

### Stability and derivatization of THC and THCA

As discussed, Δ^9^-THCA and Δ^9^-THC readily oxidize into CBNA and CBN in the presence of oxygen and light during thermal decarboxylation or even just upon aging (Fig. 6c) (Moreno-Sanz 2016; Pellati et al., 2018; Dussy et al. 2005) in the same way, during storage or during decarboxylation, Δ^9^-THC can also oxidize into an isomer known as Δ^8^-THC, which is an artifact of the aging process (Pellati et al., 2018). As decarboxylation is only partial, THCA can be found, together with Δ^9^-THC, in the oral fluid, serum, and urine of cannabis consumers (Dussy et al. 2005; Jung et al. 2007; Moore et al. 2007). This can be used forensically, as THCA does not convert to Δ^9^-THC in vivo, displaying its own metabolic and elimination pathways (Fig. 6c); consequently, the presence of THCA distinguishes between the use of plant-based cannabis and prescribed synthetic Δ^9^-THC, e.g., Marinol® (Jung et al. 2009; Raikos et al. 2014). Although still relevant in jurisdictions practicing prohibition, this is likely to become far less important as legalization spreads.

In 1970, Agurell et al. confirmed the existence of acid metabolites of Δ^9^-THC (Agurell et al. 1970). The authors injected radiolabeled Δ^9^-THC into rabbits; urine analysis confirmed the presence of 11-nor-9-carboxy-delta 9-THC (THC-COOH, Fig. 6c). THC-COOH produces no psychotropic responses in humans, and is further metabolized into glucuronide conjugates (Wall and Perez-Reyes 1981). THC-COOH does not elicit cannabimimetic behaviors in mice and shows no affinity for the CB1 receptor (Martin et al. 1995). A related carboxylate derivative of THC (carboxylic acid analog of Δ^9^-THC, Fig. 6c) was isolated from high potency *C. sativa* plants (Husni et al. 2014). This compound, improperly referred to as Δ^9^-THC, displayed low affinity (in the mM range) for both CB1 and CB2 receptors. This is in agreement with a previous report, where Δ^9^-THCA was synthesized as part of a structure–activity relationship study conducted on the C-1 position of Δ^9^-THC (Burdick et al. 2010). The analysis of the metabolites of the other cannabinoids has not been extensively studied and could prove fruitful; however, the low affinity of carboxylated cannabinoids for their receptors likely implies that they will be inactive on this pathway.

### Concluding remarks and future perspectives

Although much effort has been expended to investigate the biosynthesis of cannabinoids, and their mechanisms of decarboxylation and metabolism, much remain unclear. We still lack structural data for many of the enzymes involved, and we have little information about how the approximately 200 different cannabinoids are each prepared. At a larger scale, the relative role and gene expression profile of the different trichomes in the plant is not understood. Research into the physiological activity of cannabis has been largely restricted to THC and CBD, but there is clear evidence that some of the effects arise from the other cannabinoids. As their promise for therapeutics becomes ever clearer, we will need a better understanding of these pathways so that we can re-engineer them, either in the plant or a recombinant vector, for their selective production. Remarkable progress has been achieved in the last two decades in cannabinoid natural product chemistry, but much work remains to be done to attain the goal of producing chosen cannabinoids in high quantities and purity for both therapeutic and recreational purposes.

## Supplementary Information

**Additional file 1:** **Table S1.** Classification of major cannabinoids based on their chemical nature discovered from *C. sativa* L. and change in their numbers from 2005 to 2015. **Figure S1.** The three types of glandular trichomes found on female cannabis flowers. (a) magnified trichomes from the female plant, (b) schematic illustration of stalked trichome showing different regions and storage of cannabinoids, (c-e) cryo-SEM images of the three types of cannabis glandular trichomes, classified as stalked (c), sessile (d), and bulbous (e); scale bars 20 um. Panels c-e are reproduced from Livingston et al. 2020.^1^ Panel A is reproduced under a creative commons licence. **Figure S2.** HPLC chromatograms recorded at 220 nm for the conversion of Δ9-THCA to Δ9-THC at different temperatures by Dussy and coworkers. At higher temperatures (160 °C, 180 °C, Δ9-THC is oxidised to form cannabinol, Reproduced from Dussy et al. 2005.^6^ **Figure S3.** (a) UHPSFC/PDA (220 nm) chromatogram of a mixture of cannabinoid standards. Peak assignment: (1) CBD, (2) Δ^8^-THC, (3) THCV, (4) Δ^9^-THC, (5) CBN, (6) CBG, (7) THCA, (8) CBDA, (9) CBGA (see list of abbreviations at the end of the article for explanation, (b) concentration (mM) of THCA, (c) Δ^9^-THC (d) CBDA and (e) CBD as function of time and temperature. Figures reproduced from Wang et al. 2016.^8^ **Figure S4.** Decarboxylation of 2-hydroxybenzoic acid via a β-keto acid intermediate as proposed by Perrotin-Brunel.^9^
