# Correction: The biosynthesis of the cannabinoids

**DOI:** 10.1186/s42238-025-00292-w

**Published:** 2025-07-22

**Authors:** M. Nazir Tahir, Farsheed Shahbazi Raz, Simon Rondeau-Gagné, John F. Trant

**Affiliations:** Department of Chemistry and Biochemistry, University of Windsor, 401 Sunset Avenue, Windsor, ON N9B 3P4 Canada

**Correction: J Cannabis Res 3, 7 (2021).**

10.1186/s42238-021-00062-4.

Following the publication of the original article, the author reported that the author name “**Fred Shahbazi**” should be updated to reflect the legal and professional name: “**Farsheed Shahbazi Raz.**”

This has been updated above and the original article (Tahir et al. 2021) has been corrected.
