# Supplementary material for: The biosynthesis of the cannabinoids
Source: J Cannabis Res. 2021 Mar 15;3:7. doi: 10.1186/s42238-021-00062-4 (PMC7962319; doi:10.1186/s42238-021-00062-4)
Supplement: Supplementary file 1 — Additional file 1: Table S1. Classification of major cannabinoids based on their chemical nature discovered from C. sativa L. and change in their numbers from 2005 to 2015. Figure S1. The three types of glandular trichomes found on female cannabis flowers. (a) magnified trichomes from the female plant, (b) schematic illustration of stalked trichome showing different regions and storage of cannabinoids, (c-e) cryo-SEM images of the three types of cannabis glandular trichomes, classified as stalked (c), sessile (d), and bulbous (e); scale bars 20 um. Panels c-e are reproduced from Livingston et al. 2020.1 Panel A is reproduced under a creative commons licence. Figure S2. HPLC chromatograms recorded at 220 nm for the conversion of Δ9-THCA to Δ9-THC at different temperatures by Dussy and coworkers. At higher temperatures (160 °C, 180 °C, Δ9-THC is oxidised to form cannabinol, Reproduced from Dussy et al. 2005.6 Figure S3. (a) UHPSFC/PDA (220 nm) chromatogram of a mixture of cannabinoid standards. Peak assignment: (1) CBD, (2) Δ8-THC, (3) THCV, (4) Δ9-THC, (5) CBN, (6) CBG, (7) THCA, (8) CBDA, (9) CBGA (see list of abbreviations at the end of the article for explanation, (b) concentration (mM) of THCA, (c) Δ9-THC (d) CBDA and (e) CBD as function of time and temperature. Figures reproduced from Wang et al. 2016.8 Figure S4. Decarboxylation of 2-hydroxybenzoic acid via a β-keto acid intermediate as proposed by Perrotin-Brunel.9 [file 42238_2021_62_MOESM1_ESM.docx]

**Supporting Information for:**

**The Biosynthesis of the Cannabinoids**

M. Nazir Tahir, Fred Shahbazi, Simon Rondeau-Gagné* and John F. Trant*

*Department of Chemistry and Biochemistry, University of Windsor, 401 Sunset Avenue, Windsor, Ontario N9B 3P4, Canada*

*Corresponding authors. Tel.: +1 519 253 3000 x3556 (S. Rondeau-Gagn*é*), x3528 (J.F. Trant)*

*fax: +1 519 973 7098*

*E-mail addresses: srondeau@uwindsor.ca (S. Rondeau-Gagn*é*), jtrant@uwindsor.ca (J.F. Trant)*

| **Table 1**: Classification of major cannabinoids based on their chemical nature discovered from *C. sativa* L. and change in their numbers from 2005 to 2015 | | |
| --- | --- | --- |
| Chemical class | Number discovered in: | |
|  | 2005 | 2015 |
| Δ^9^-THC type | 9 | 23 |
| Δ^8^-THC type | 2 | 5 |
| CBG type | 8 | 16 |
| CBC type | 6 | 9 |
| CBD type | 7 | 7 |
| CBND type | 2 | 2 |
| CBE type | 5 | 5 |
| CBL type | 3 | 3 |
| CBN type | 7 | 11 |
| CBT type | 9 | 9 |
| Miscellaneous | 14 | 30 |
| Total cannabinoids | 72 | 120 |
| Total non-cannabinoids | 419 | 445 |
| Total chemical compounds | 491 | 565 |


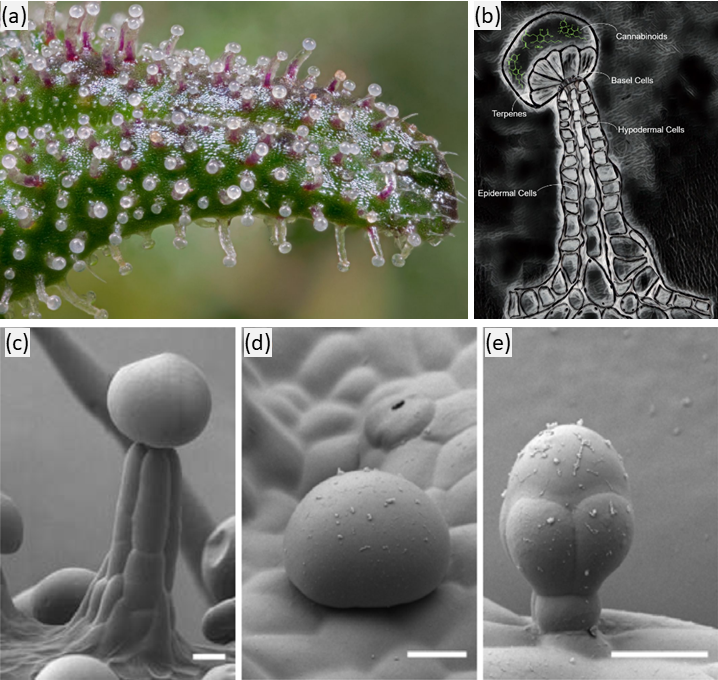


**Figure S1.** The three types of glandular trichomes found on female cannabis flowers. (a) magnified trichomes from the female plant, (b) schematic illustration of stalked trichome showing different regions and storage of cannabinoids, (c-e) cryo-SEM images of the three types of cannabis glandular trichomes, classified as stalked (c), sessile (d), and bulbous (e); scale bars 20 um. Panels c-e are reproduced from Livingston *et al.* **2020**.^1^ Panel A is reproduced under a creative commons licence.\

**Analytical Tools for Cannabis Analysis.** GC is a relatively simple technique for studying decarboxylation and is ideal in most of the cases specially for low molecular weight (280–360 gmol^-1^) cannabinoids; however its major limitation involves its necessary heating of the sample, therefore, it can not be used for quantifying labile acids as they decarboxylate under the conditions.^2^ High Performance Liquid Chromatography (HPLC) is effective for both classes but has its own set of drawbacks. Raharjo and Verpoorte reviewed different HPLC reported methods for cannabinoid analysis and concluded that most were neither validated nor compatible with regulations, and that many did not provide clean separation of the major cannabinoids without overlapping peaks.^3^ To overcome the drawbacks of each technique, Hazekamp and coauthors propose combining HPLC with a secondary analysis by GC as a method to quantify both the carboxylated and decarboxylated forms of all major cannabinoids in a single run.^4^ Coupling of mass spectrometry (MS) with HPLC or GC can also be used resolve all peaks in a single analysis, however MS is not routinely available in all industrial and academic laboratories.

**Detailed discussion regarding the mechanism of decarboxylation.** To study the decarboxylation of **CBDA** and **THCA**, Veress *et al*. heated dried extracts in an open reactor over a range of temperatures and times and analyzed the samples by HPLC.^5^ Results indicate that complete decarboxylation of **Δ^9^-THCA** into **Δ^9^-THC** was achieved in 5–10 minutes at 145°C. Longer heating times caused a significant loss, possibly due to the evaporation of **Δ^9^-THC** in open reactor, and from side reactions.^5^ To minimize this loss caused by longer heating, Dussy and coworkers heated pure **THCA** in an oven for a fixed time of 15 minutes at 120°C, 140°C, 160°C, and at 180°C (Figure 5).^6^ Decarboxylation progressed at 160°C; however, formation of an oxidation product, cannabinol (**CBN**), was observed at 160°C and 180°C (Figure 5). In this study,^6^ the molar sum of **Δ^9^-THC** and **THCA**, measured by HPLC, was always higher than **Δ^9^-THC** as measured by GC, indicating incomplete decarboxylation and conversion never exceeding 70%. The authors suggested this discrepancy arose from the formation of degraded polymeric material during thermal decarboxylation based on the fact that the sum of **Δ^9^-THCA**, **Δ^9^-THC** and cannabinol (**CBN**) never reaches 100% of the original value.^6^ In the same report, they used GC to study the decarboxylation of **Δ^9^-THCA** *in situ*, using the heat of the instrument to initiate decarboxylation, mimicking smoking conditions. However, yields were moderate, with conversion peaking at 65% at 225 ºC, their best set of conditions: at higher temperatures (e.g. 300°C), yields fell precipitously. In contrast, Hewavitharana *et al*.^7^ reported a complete decarboxylation of the acids to neutral cannabinoids under relatively simple conditions: CBD-rich leaves were dried at 40°C followed by extraction with hexane. The extract was analyzed by GC-MS using a heated injection port and crysenene-d12 as an internal standard. They obtained a linear calibration range extending as high as 40 ppm and a detection limit of 0.2 ng/injection. Under these conditions, conversion of **CBDA** into **CBD** was complete and no degradation was noted; however, all these methods share the significant limitation that GC injection-mediated decarboxylation is unsuitable for production-scale.

Although HPLC is the preferred method to study cannabinoids and their decarboxylation, a major challenge is their lack of chromophores which means they have low molar absorptivity. UV-vis analysis is restricted low wavelengths, where the strong background absorbance from the eluant results in poor detection limits. However, this problem can be overcome by replacing UV-detection with mass spectrometry (HPLC-MS). This was the approach of Wang *et al*., who quantitatively determined nine cannabinoids (**CBD**, **Δ^8^-THC**, **THCV**,**Δ^9^-THC**, **CBN**, **CBG**, **THCA**, **CBDA** and **CBGA**) before and after cannabis extracts were heated in a vacuum oven at 80°C, 95°C, 110°C, 130°C and 145°C for up to 60 minutes (Figure S3).^8^ All cannabinoids in this study were analyzed by ultra-high performance supercritical fluid chromatography/photodiode array-mass spectrometer (UHPSFC/PDA-MS) before and after decarboxylation (Figure S3). Decarboxylation at different temperatures displayed an exponential relationship between concentration and time indicating a first-order or pseudo-first-order reaction as would be expected for the unimolecular process of spontaneous aromatic decarboxylation. The decarboxylation rate constant for **Δ^9^-THCA** was determined to be twice that of either **CBDA** or **CBGA**. **THCA** decarboxylation was nearly quantitative with no side reactions detected (8% loss to evaporation, see Figure 6). No CBN, a common oxidation by-product, (Scheme 7) was observed under the experimental conditions. In contrast, decarboxylation reactions for **CBDA** and **CBGA** were more complex with undetermined side reactions accounting for a loss of 18% (**CBDA**) to 53% (**CBGA**) of the extract mass.^8^

The kinetics of cannabinoid decarboxylation is of critical importance for industrial processing. Veress and coauthors reported the kinetics of decarboxylation as a function of temperature, solvents, sorbent phases and additives in 1990.^5^ However, the results are only semi-quantitative due to evaporation and degradation of the analytes. Perrotin-Brunel led a team that revisited this question in 2011, combining HPLC analysis with molecular modelling to measure the rates of **THCA** decarboxylation in the solid state.^9^ Cannabis extract was blended in a mixer, then heated from 90°C to 140°C under vacuum. Aliquots were collected every 5 min for the first hour and then every half an hour until the conversion of **Δ^9^-THCA** to **Δ^9^-THC** was complete. Under the experimental conditions, the highest conversion and yield to **Δ^9^-THC** was obtained at 110 °C after 110 min, which is consistent with the qualitative findings of Veress *et al*.^5^ The solid-state reaction obeys a first order rate law, as expected. The highlight of this work was that it was the first attempt to study kinetics and decarboxylation in real-time, in contrast to most of the previous work that was conducted either in closed reactors, which don’t allow sampling,^10-11^ or in open reactors and on a glass surface, that caused loss of material.^5^ The authors proposed pseudo-first-order, acid catalyzed keto–enol mechanism (Scheme 8) for the decarboxylation process; the computational calculations are consistent with this mechanism. For simplicity, both the experimental and computational studies were performed using 2-hydroxybenzoic acid (Scheme **S4**); indicating that this might be a general feature of 2-hydroxybenzoic acids.


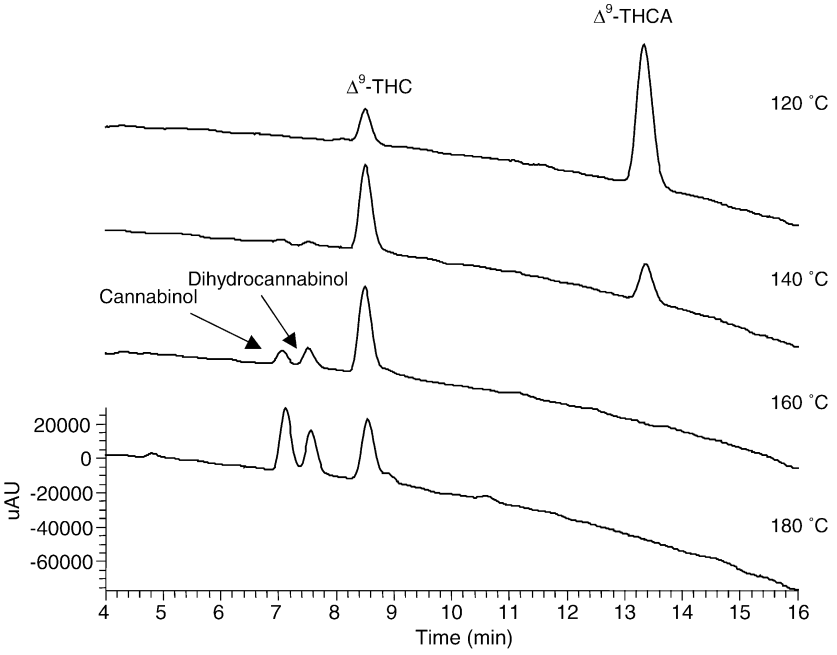


**Figure S2**: HPLC chromatograms recorded at 220 nm for the conversion of Δ9-THCA to Δ9-THC at different temperatures by Dussy and coworkers. At higher temperatures (160 °C, 180 °C, Δ9-THC is oxidised to form cannabinol, Reproduced from Dussy *et al.* **2005**.^6^


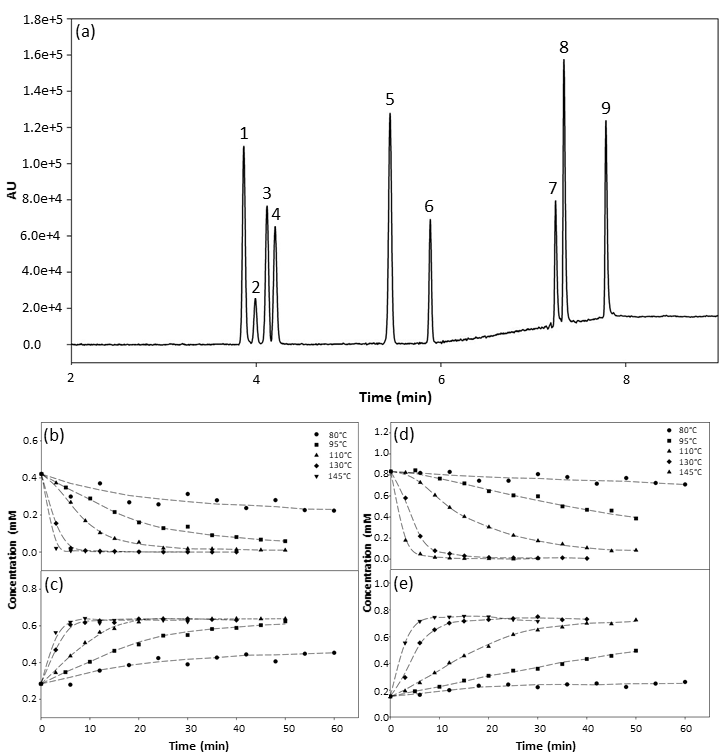


**Figure S3**: (a) UHPSFC/PDA (220 nm) chromatogram of a mixture of cannabinoid standards. Peak assignment: (1) **CBD**, (2) **Δ^8^-THC**, (3) **THCV**, (4) **Δ^9^-THC**, (5) **CBN**, (6) **CBG**, (7) **THCA**, (8) **CBDA**, (9) **CBGA** (see list of abbreviations at the end of the article for explanation, (b) concentration (mM) of **THCA**, (c) **Δ^9^-THC** (d) **CBDA** and (e) **CBD** as function of time and temperature. Figures reproduced from Wang *et al.* **2016**.^8^

**Figure S4**: Decarboxylation of 2-hydroxybenzoic acid via a β-keto acid intermediate as proposed by Perrotin-Brunel.^9^

1. Livingston, S. J.; Quilichini, T. D.; Booth, J. K.; Wong, D. C. J.; Rensing, K. H.; Laflamme-Yonkman, J.; Castellarin, S. D.; Bohlmann, J.; Page, J. E.; Samuels, A. L., Cannabis glandular trichomes alter morphology and metabolite content during flower maturation. *Plant J.* **2020,** *101* (1), 37-56.

2. De Backer, B.; Debrus, B.; Lebrun, P.; Theunis, L.; Dubois, N.; Decock, L.; Verstraete, A.; Hubert, P.; Charlier, C., Innovative development and validation of an HPLC/DAD method for the qualitative and quantitative determination of major cannabinoids in cannabis plant material. *J. Chromatogr. B: Anal. Technol. Biomed. Life Sci.* **2009,** *877* (32), 4115-4124.

3. Raharjo, T. J.; Verpoorte, R., Methods for the analysis of cannabinoids in biological materials: a review. *Phytochem. Anal.* **2004,** *15* (2), 79-94.

4. Hazekamp, A.; Peltenburg, A.; Verpoorte, R.; Giroud, C., Chromatographic and spectroscopic data of cannabinoids from *Cannabis sativa L*. *J. Liq. Chromatogr. Relat. Technol.* **2005,** *28* (15), 2361-2382.

5. Veress, T.; Szanto, J. I.; Leisztner, L., Determination of cannabinoid acids by high-performance liquid chromatography of their neutral derivatives formed by thermal decarboxylation: I. Study of the decarboxylation process in open reactors. *J. Chromatogr. A* **1990,** *520*, 339-347.

6. Dussy, F. E.; Hamberg, C.; Luginbühl, M.; Schwerzmann, T.; Briellmann, T. A., Isolation of Δ9-THCA-A from hemp and analytical aspects concerning the determination of Δ9-THC in cannabis products. *Forensic Sci. Int.* **2005,** *149* (1), 3-10.

7. Hewavitharana, A. K.; Golding, G.; Tempany, G.; King, G.; Holling, N., Quantitative GC-MS analysis of Δ9-tetrahydrocannabinol in fiber hemp varieties. *J. Anal. Toxicol.* **2005,** *29* (4), 258-261.

8. Wang, M.; Wang, Y.-H.; Avula, B.; Radwan, M. M.; Wanas, A. S.; Antwerp, J. v.; Parcher, J. F.; ElSohly, M. A.; Khan, I. A., Decarboxylation study of acidic cannabinoids: A novel approach using ultra-high-performance supercritical fluid chromatography/photodiode array-mass spectrometry. *Cannabis Cannabinoid Res.* **2016,** *1* (1), 262-271.

9. Perrotin-Brunel, H.; Buijs, W.; Spronsen, J. v.; Roosmalen, M. J. E. v.; Peters, C. J.; Verpoorte, R.; Witkamp, G.-J., Decarboxylation of Δ9-tetrahydrocannabinol: Kinetics and molecular modeling. *J. Mol. Struct.* **2011,** *987* (1), 67-73.

10. Kanter, S. L.; Musumeci, M. R.; Hollister, L. E., Quantitative determination of Δ9-tetrahydrocannabinol and Δ9-tetrahydrocannabinolic acid in marihuana by high-pressure liquid chromatography. *J. Chromatogr.* **1979,** *171*, 504-8.

11. Smith, R. N.; Vaughan, C. G., High-pressure liquid chromatography of cannabis. Quantitative analysis of acidic and neutral cannabinoids. *J. Chromatogr.* **1976,** *129*, 347-54.
